# EAST MEETS WEST: INNOVATIVE APPROACHES TO GERONTOLOGY EDUCATION AND TRAINING

**DOI:** 10.1093/geroni/igad104.1090

**Published:** 2023-12-21

**Authors:** Megumi Inoue, Takashi Amano, Nancy Morrow-Howell

## Abstract

The global population is aging at an increasingly fast pace compared to past generations. Although the magnitudes and rates of aging in the United States and Japan differ, the drastic change in population structure has resulted in common challenges for both countries. One challenge is preparing people and society for living a long life while promoting well-being in old age for everyone. Combating ageism, delivering accurate information about aging, and encouraging people to have a balanced view of the aging process are all important topics for living in an aging society. Therefore, gerontology education and training plays a critical role. In this symposium, we will present examples of current efforts and research findings on aging education and training in both the United States and Japan. The first presenter will discuss their efforts on promoting gerontology education through redesigning communities with interdisciplinary collaboration in Japan. The second presenter will explain gerontology curriculum and degree programs in Japan. The third presenter will introduce an international gerontology education course designed for United States graduate students. The last presenter will report findings from a scoping review on intergenerational activities in higher education. Implications for future gerontology education will also be discussed.

